# Modulation of Fibroblast Phenotype by Colorectal Cancer Cell-Secreted Factors Is Mostly Independent of Oncogenic KRAS

**DOI:** 10.3390/cells11162490

**Published:** 2022-08-11

**Authors:** Patrícia Dias Carvalho, Susana Mendonça, Flávia Martins, Maria José Oliveira, Sérgia Velho

**Affiliations:** 1 Instituto de Investigação e Inovação em Saúde (i3S), Rua Alfredo Allen 208, 4200-135 Porto, Portugal; 2 Institute of Molecular Pathology and Immunology of the University of Porto (IPATIMUP), Rua Júlio Amaral de Carvalho 45, 4200-135 Porto, Portugal; 3 Institute of Biomedical Sciences Abel Salazar (ICBAS), University of Porto, Rua Jorge de Viterbo Ferreira 228, 4050-313 Porto, Portugal; 4 Department of Pathology, Faculty of Medicine of the University of Porto (FMUP), Alameda Prof. Hernâni Monteiro, 4200-319 Porto, Portugal; 5 Institute of Biomedical Engineering (INEB), University of Porto, Rua Alfredo Allen 208, 4200-135 Porto, Portugal

**Keywords:** cancer-associated fibroblasts, colorectal cancer, mutant KRAS, secreted factors

## Abstract

KRAS mutations have been shown to extend their oncogenic effects beyond the cancer cell, influencing the tumor microenvironment. Herein, we studied the impact of mutant KRAS on the modulation of the pro-tumorigenic properties of cancer-associated fibroblasts (CAFs), including α-SMA expression, TGFβ1 and HGF production, extracellular matrix components and metalloproteinases expression as well as collagen contraction and migration capacities. To do so, CCD-18Co normal-like colon fibroblasts were challenged with conditioned media from control and KRAS silenced colorectal cancer (CRC) cells. Our results showed that the mutant KRAS CRC cell-secreted factors were capable of turning normal-like fibroblasts into CAF-like by modulating the α-SMA expression, TGFβ1 and HGF production and migration capacity. Oncogenic KRAS played a secondary role as its silencing did not completely impair the capacity of CRC cells to modulate most of the fibroblast properties analyzed. In summary, our work suggests that mutant KRAS does not play a major role in controlling the CRC cell-secreted factors that modulate the behavior of fibroblasts. The fact that CRC cells retain the capacity to modulate the pro-tumorigenic features of fibroblasts independently of KRAS silencing is likely to negatively impact their response to KRAS inhibitors, thus standing as a putative mechanism of resistance to KRAS inhibition with potential therapeutical relevance.

## 1. Introduction

KRAS is the oncogene most frequently mutated in cancer and features in the group of key cancer targets. Notwithstanding that mutations were discovered nearly 40 years ago, it remains one of the few that still lacks efficient therapies [1]. Mutations occur in numerous cancer types at variable frequencies, prevailing in lung (32%), colorectal (CRC; 50%) and pancreatic (88%) cancers [2]. They render KRAS constitutively active, thus fueling the cancer cell with strong mitogenic, survival, stemness, invasive and pro-metastatic signals [3,4]. Adding to this major oncogenic role, mutant KRAS (mutKRAS) is also a predictive factor of resistance to anti-EGFR-targeted therapies [5].

In addition to the role of mutKRAS in controlling key cancer activities, it has been shown that its oncogenic signaling extends beyond the cancer cell to orchestrate a pro-tumorigenic microenvironment [6,7,8,9,10,11]. Within the tumor microenvironment (TME), immune cells and fibroblasts are amongst the most commonly found cell types [6,12]. The effect of mutKRAS on the regulation of the tumor immune microenvironment is one of the most well-described non-cancer cell effects. Specifically, mutKRAS immunomodulatory effects have been reported at the level of recruitment, activation and differentiation of immune cells, supporting the pro-tumorigenic role of the immune compartment as well as at the cancer cell level, promoting evasion from immunosurveillance [6,7,8,9,10,11,13]. The effects of mutKRAS on the modulation of fibroblasts and cancer-associated fibroblasts (CAFs) phenotypes are not well-studied and the majority of the available studies focus on pancreatic cancer [6,7]. Still, the existing data suggest that KRAS mutations in cancer cells can alter the behavior of fibroblasts, which in turn affect both the cancer cells and the microenvironment through extracellular matrix (ECM) changes and growth factor signaling, contributing to tumor progression [6,14].

CAFs are a heterogeneous and plastic population, originating in their majority from the “activation” of local tissue fibroblasts, for instance, by the action of tumor cell-secreted factors such as transforming growth factor β (TGFβ) or platelet-derived growth factor (PDGF) [15,16]. Together with the expression of several markers such as α-smooth muscle actin (α-SMA) and fibroblast-activation protein-α, this activation state foresees the acquisition of contraction, proliferation and ECM synthesis/remodeling capacities as well as a highly secretory phenotype [15]. These characteristics make CAFs major modulators of the TME [15,16]. They participate in the acquisition and maintenance of most cancer hallmarks, such as sustaining proliferative signaling, deregulating cellular energetics, activating invasion and metastasis and inducing angiogenesis, and are active players in the construction of an immune permissive environment that supports cancer progression [12,17]. In CRC, CAFs represent the most abundant stromal population [12]. They are found at the tumor invasive front [18,19] and their abundance is a poor prognosis factor [20]. Hence, given the central roles of CAFs in CRC progression and malignancy, studies approaching CRC cell–fibroblasts crosstalk are still needed. Moreover, a better understanding of how mutKRAS affects the TME may reveal potential therapeutic targets to impair mutKRAS cancer cells by reverting the pro-tumorigenic properties of the TME components.

Herein, we aimed to uncover the effect of secreted factors from mutKRAS CRC cells on the modulation of fibroblast properties. Our data revealed that fibroblast properties were modulated by the cancer cell-secreted factors, mostly independently of KRAS and in a cell line-dependent context. Therefore, our work sheds light on a potential mechanism of resistance to KRAS inhibition: upon KRAS inhibition, cancer cells can continue to promote pro-tumorigenic properties on the fibroblasts, which in turn may support the survival of cancer cells through KRAS-independent mechanisms.

## 2. Materials and Methods

### 2.1. Cell Culture

HCT116 CRC cell line and CCD-18Co normal colon fibroblasts were purchased from the American Type Culture Collection. The LS174T CRC cell line was kindly provided by Dr. Ragnhild A. Lothe (Oslo University Hospital, Oslo, Norway).

HCT116 was cultured in RPMI 1640 medium (Gibco, Thermo Fisher Scientific, Waltham, MA, USA); LS174T and CCD-18Co were cultured in DMEM medium (Gibco, Thermo Fisher Scientific, Waltham, MA, USA). For all cell lines, the respective medium was supplemented with 10% fetal bovine serum (HyClone, Logan, UT, USA) and 1% penicillin–streptomycin (Gibco, Thermo Fisher Scientific, Waltham, MA, USA). The cells were maintained at 37 °C in a humidified atmosphere with 5% CO_2_.

### 2.2. Gene Silencing with siRNA and Conditioned Medium Production

HCT116 (150,000 cells/well) and LS174T (200,000 cells/well) were seeded in six-well plates and transfected after approximately 16 h. The transfection was performed using Lipofectamine RNAiMAX (Invitrogen, Thermo Fisher Scientific, Waltham, MA, USA) in a reduced-serum Opti-MEM medium (Gibco, Thermo Fisher Scientific, Waltham, MA, USA), following the manufacturer’s recommendations. The silencing of total KRAS was achieved using a specific ON-TARGETplus SMARTpool small interfering RNA (L-005069-00-0010; Dharmacon, Lafayette, CO, USA) at a final concentration of 10 nM (for which we have previously demonstrated the impact on KRAS-driven signaling and functional effects [21,22]). A non-targeting siRNA (D-001810-01-50; ON-TARGETplus Non-targeting siRNA #1; Dharmacon) was used as a negative control. Forty-eight hours after the transfection, the cell monolayers were washed twice, and the respective serum-free culture medium was added. After an additional 24 h, the conditioned medium (CM) from all the conditions was harvested, centrifuged to remove the cell debris, filtered through a 0.2 μm filter and stored at −20 °C until use. The total protein was extracted and KRAS silencing efficiency was evaluated by Western blot (Figure S1).

### 2.3. CCD-18Co Treatment with the Conditioned Medium

The CCD-18Co fibroblasts (100,000 cells/well) were seeded in six-well plates. When approximately a 90% confluence was reached (time 0), the cells were washed twice and the CM from HCT116 and LS174T control (siCTRL) and KRAS silenced (siKRAS) cells was added. At the same time, DMEM + 1% penicillin/streptomycin and DMEM + 1% penicillin/streptomycin + 10 ng/mL rhTGFβ1 (Immunotools, GmbH, Friesoythe, Germany) were used as the negative and positive controls, respectively. After four days in culture, the subsequent experiments were performed. The media from all conditions were collected, centrifuged to remove the cell debris and stored at −20 °C. Total protein and mRNA were extracted from each condition.

### 2.4. Protein Extraction and Western Blotting

Total protein was extracted using ice-cold RIPA lysis buffer (50 mM Tris HCl; 150 mM NaCl; 2 mM EDTA; 1% IGEPAL CA-630; pH = 7.5) supplemented with a protease inhibitor cocktail (Roche, Basel, Switzerland) and a phosphatase inhibitor cocktail (Sigma-Aldrich, St. Louis, MO, USA). The protein concentration was determined using a DCProtein assay kit (Bio-Rad, Hercules, CA, USA). Briefly, 15 µg of protein were resolved on sodium dodecyl sulphate-polyacrylamide gel electrophoresis (SDS-PAGE) under denaturing conditions and transferred to Amersham Protran Premium NC 0.45 µm membranes (Cytiva, Buckinghamshire, UK). The membranes were blocked for 1 h at room temperature (RT) and incubated overnight at 4 °C with agitation, with the respective primary antibody (KRAS: LS-Bio, LS-C175665, 1:4000; α-SMA: Abcam, ab7817, 1:250; GAPDH: Santa Cruz Biotechnology, sc-47724, 1:10,000) diluted in 5% non-fat milk in PBS + 0.5% Tween 20 (Sigma-Aldrich, St. Louis, MO, USA). Subsequently, the membranes were incubated for 1 h at RT with an anti-mouse HRP-conjugated secondary antibody (Amersham ECL HRP-Conjugated- NA931, Cytiva, Buckinghamshire, UK) and the bands were detected using ECL (Bio-Rad, Hercules, CA, USA) and Amersham Hyperfilm (Cytiva, Uppsala, Sweden) exposure. The band intensity was quantified using Fiji software.

### 2.5. mRNA Expression Analysis by qRT-PCR

Total RNA was extracted using TripleXtractor (Grisp Research Solutions, Porto, Portugal), following the manufacturer’s instructions. Briefly, the cells were lysed in the TripleXtractor and, after aqueous and organic phase separation using chloroform (Merck, Darmstadt, Germany), the RNA was precipitated using isopropanol (Fisher Chemical, Loughborough, UK). After 2 rounds of washes with 70% ethanol (Fisher Chemical, Loughborough, UK), the RNA was eluted in ultrapure RNase/DNase-free water (Invitrogen, Thermo Fisher Scientific, Waltham, MA, USA). A total of 0.5 μg of RNA was converted to cDNA using qScript™ cDNA SuperMix (Quantabio, Beverly, MA, USA), according to the provided instructions. Quantitative real-time PCR (qRT-PCR) was performed using a 7500 Fast Real-Time PCR system (Applied Biosystems, Thermo Fisher Scientific, Warrington, UK) with TaqMan^®^ Universal PCR Master Mix, No AmpErase^®^ UNG (Applied Biosystems, Thermo Fisher Scientific, Warrington, UK) and standard TaqMan thermocycling conditions. TaqMan Gene Expression Assays (Applied Biosystems, Thermo Fisher Scientific, Warrington, UK) or PrimeTime qPCR Assays (Integrated DNA Technologies, Coralville, IA, USA), as referred in Table S1, were used. The relative mRNA expression levels of the targeted genes were normalized to the expression levels of the housekeeping gene *GAPDH* and estimated using the comparative 2^(−ΔCT)^ method.

### 2.6. Quantification of the Secreted Factors by ELISA

Following the manufacturer’s instructions, the levels of total TGFβ1 (Legend Max, BioLegend, San Diego, CA, USA) and HGF (RayBiotech, Peachtree Corners, GA, USA) were quantified by ELISA in the CM from HCT116 and LS174T cells (siCTRL and siKRAS) as well as in the media from all conditions collected at the final timepoint of the experiment with the CCD-18Co fibroblasts. 

### 2.7. Wound-Healing Assay

When CCD-18Co fibroblasts formed a confluent monolayer, a wound was made using a 200 µL tip. The detached cells were washed and controls and CM from HCT116 and LS174T siCTRL and siKRAS cells were added to the respective well. Two technical replicates/conditions were performed. The wound healing was monitored every 24 h until the final timepoint of four days. At all timepoints, 2 areas/wound were photographed using a Leica DMi1 inverted microscope with a camera, using the 5 × objective. The wound area was quantified using Fiji software and the percentage of wound closure over time was calculated. 

### 2.8. Collagen Contraction Assay

After four days in culture, CCD-18Co fibroblasts were trypsinized, washed and a cell suspension of 3 × 10^5^ cells/mL was prepared in the appropriate control media or CM. From this cell suspension, 400 µL was mixed with 200 µL of 3 mg/mL collagen solution in 0.1% acetic acid (rat tail type I; Merck Millipore, Burlington, MA, USA), followed by the addition of 10 μL of 1 M NaOH. As cell-free negative controls, 400 µL of each medium was mixed with the collagen solution. From each mixture, 500 µL was plated in a 24-well plate and the gels were allowed to solidify at RT for 1 h. After solidification, 600 µL of the respective serum-free control media or CM was added and the gels were completely dissociated from the wells by gently running a pipette tip along the gel edges. The plate was maintained in optimal culture conditions for 8 h. The images of the plate were taken in a Bio-Rad Gel-Doc Imager and the final area of each gel was measured using Fiji software.

### 2.9. Statistical Analysis

The statistical analyses were performed using GraphPad Prism software (version 9.0.0, La Jolla, CA, USA). The results were expressed as mean ± standard deviation (SD). The specific performed test as well as the number of independent biological replicates are referred on each figure.

## 3. Results

### 3.1. CRC Cell-Secreted Factors Influence the Expression of α-SMA and the Secretion of TGFβ1 and HGF by CCD-18Co Fibroblasts

**Figure 1:**
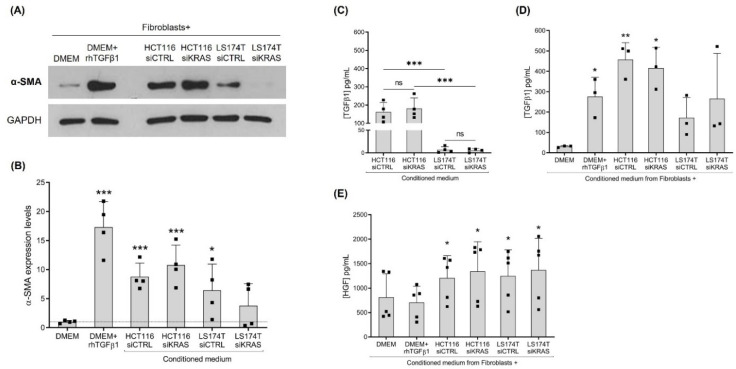
**Culture with conditioned media (CM) from CRC cells is capable of influencing the expression levels of α-SMA and the production of TGFβ1 and HGF by CCD-18Co fibroblasts.** (**A**) Representative Western blot illustrating α-SMA expression levels; (**B**) the respective quantification of four independent biological replicates showing values normalized to the DMEM control. Levels of total TGFβ1 in the: (**C**) CM from HCT116 and LS174T siCTRL and siKRAS cells, and (**D**) CM collected from fibroblasts upon four days of culture with control media (DMEM and DMEM+rhTGFβ1) and CM of HCT116 and LS174T (siCTRL and siKRAS) cells. (**E**) Levels of HGF produced by fibroblasts upon four days of culture with control media (DMEM and DMEM+rhTGFβ1) and CM of HCT116 and LS174T (siCTRL and siKRAS) cells. Values of the independent experiments (represented as black squares) were plotted as mean ± SD. Data normality was tested using the Shapiro–Wilk test and a parametric or non-parametric test was performed in accordance: *t*-test comparing all conditions with the DMEM control (**B**,**D**,**E**); and one-way ANOVA with Šídák’s multiple comparison test (**C**) (* *p* ≤ 0.05; ** *p* ≤ 0.01; *** *p* ≤ 0.001; ns: not significant).

In order to evaluate if oncogenic KRAS signaling extended to the outside of the cancer cells affecting the properties of the fibroblasts, we evaluated the fibroblast activation state in response to cancer cell-secreted factors. To do so, CCD-18Co fibroblasts were treated with the CM from the CRC cells, siCTRL and siKRAS, as well as the control media (with and without rhTGFβ1). After four days of treatment, total protein was extracted and the expression of the fibroblast-activation marker α-SMA was evaluated. As expected, the rhTGFβ1 treatment resulted in a significant increase in α-SMA protein expression. Similarly, the CM from HCT116 cells was able to increase the expression levels of α-SMA, an effect that was independent of KRAS silencing. Even though to smaller levels, the CM from the LS174T siCTRL cells were also capable of increasing the expression of this marker, whereas the CM from LS174T siKRAS failed to reach a statistical significance (Figure 1A, B). Attempting to explain these results, total TGFβ1 was quantified in the CM from CRC cells. The results showed that both HCT116 and LS174T cells secreted similar levels of TGFβ1 when comparing siCTRL with the siKRAS conditions. Notably, and in accordance with the levels of activation observed, the levels of TGFβ1 were higher in HCT116-derived CM in comparison with the LS174T-derived CM (Figure 1C). Moreover, challenging fibroblasts with CM resulted in a significant increase in TGFβ1 levels upon treatment with HCT116 siCTRL and siKRAS CM, whereas the CM from LS174T cells showed no significative effect (Figure 1D).

Previous work from our group [22] and others [23] has demonstrated that HGF is an important fibroblast-secreted pro-invasive factor. HGF has also been reported to induce the activation of fibroblasts, supporting their tumor-promoting characteristics [24]. Hence, the levels of this growth factor were evaluated in the CM from the siCTRL and siKRAS CRC cells and in the CM from the treated fibroblasts as well. Quantification by ELISA showed that HGF was not detected in the CM from CRC cells. It was only detected in the fibroblast cultures and, in accordance with our previous results [22], the non-activated and rhTGFβ1-activated fibroblasts produced similar levels of HGF. Notably, challenging of fibroblasts with CM from CRC cells (both siCTRL and siKRAS) resulted in a significant increase in HGF production when compared with the non-activated fibroblasts (DMEM control) (Figure 1E).

Together, this set of results demonstrates that the impact of the CRC cell-secretome on fibroblasts activation (α-SMA expression) and on TGFβ1 and HGF secretion were mostly independent of oncogenic KRAS except for LS174T, in which the α-SMA expression demonstrated a KRAS dependency.

### 3.2. CRC Cell-Secretome Has No Influence on the mRNA Expression of Extracellular Matrix Components, but Alters the Expression of MMPs

**Figure 2:**
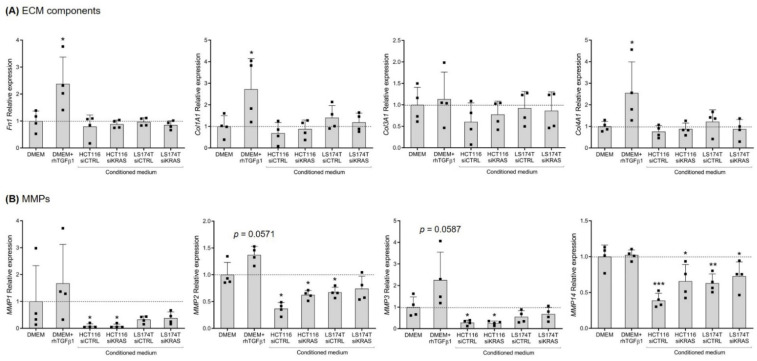
**Conditioned media from CRC cells have no impact on fibroblast expression of ECM components, but show some effects on the expression of MMPs.** (**A**) Relative expression levels of ECM components *Fn1*, *Col1A1*, *Col3A1* and *Col4A1*, and(**B**) *MMPs 1*, *2*, *3* and *14*. Values of the independent experiments (represented as black squares) were normalized to the DMEM control and were plotted as mean ± SD. Data normality was tested using the Shapiro–Wilk test and a parametric or non-parametric *t*-test was performed in accordance, comparing all conditions with the DMEM control (* *p* ≤ 0.05; ** *p* ≤ 0.01; *** *p* ≤ 0.001).

As a major role of fibroblasts is the production and remodeling of the ECM, the expression of several ECM components—fibronectin I; collagen type I, III and IV; and MMP1, 2, 3, 9 and 14—was evaluated in the total mRNA from the fibroblasts. Regarding the ECM components, the qRT-PCR results showed that activation with rhTGFβ1 resulted in a significative upregulation of *Fn1*, *Col1A1* and *Col4A1*, having no effect on the expression of *Col3A1*. None of the CM of CRC cells showed an influence on the expression of the analyzed ECM components (Figure 2A). Concerning the expression of MMPs, rhTGFβ1 showed a tendency to increase the expression of *MMP2* and *MMP3*, with no impact on the expression of *MMP1* and *MMP14*. In opposition, the CM from HCT116 cells (both siCTRL and siKRAS) downregulated the expression of *MMP1*, *2*, *3* and *14*. The CM of LS174T siCTRL cells revealed to only downregulate *MMP2* and *14*; the CM of LS174T siKRAS cells only reached a statistical significance for *MMP14,* possibly as an effect of the higher variability observed in *MMP2* (Figure 2B). The expression of *MMP9* was not detected in any of the tested conditions.

These data showed that CRC cell-secreted factors did not influence the mRNA levels of the analyzed ECM components. However, all the detected MMPs were downregulated by HCT116 cell-secreted factors independently of KRAS. The CM of LS174T cells downregulated the expression of *MMP2* and *14*, though only *MMP2* downregulation showed an association with KRAS.

### 3.3. HCT116 and LS174T Cell-Secreted Factors Differentially Affect Fibroblast Collagen Contraction and Migration Capacities

**Figure 3:**
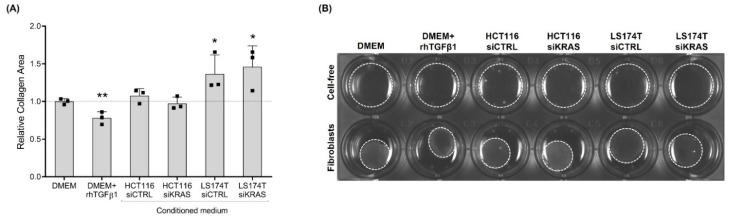
**rhTGFβ1 and conditioned media from LS174T cells are capable of influencing fibroblast contraction capacity.** After four days in culture with control media (DMEM and DMEM+rhTGFβ1) and CM of HCT116 and LS174T (siCTRL and siKRAS) cells, pre-educated fibroblasts were embedded in collagen type I and the relative area of the collagen pads was quantified after 8 h. (**A**) Collagen gels with rhTGFβ1-activated fibroblasts presented a decreased area, demonstrating an increase in fibroblast contraction capacity. CM from HCT116 cells (siCTRL and siKRAS) had no effect on collagen area. Collagen gels with CM from LS174T siCTRL and siKRAS cells showed an increased area, indicating a decreased fibroblast contraction capacity. Values of the independent experiments (represented as black squares) were normalized to the DMEM control and were plotted as mean ± SD. Data normality was tested using the Shapiro–Wilk test and a parametric or non-parametric *t*-test was performed in accordance comparing all conditions with the DMEM control (* *p* ≤ 0.05; ** *p* ≤ 0.01). (**B**) Representative image showing cell-free gels lacking contraction and contracted fibroblast-populated gels.

As contraction and migratory capacities are also important features of activated fibroblasts, we evaluated those capacities. The contraction capacity was evaluated by assessing the changes in the area of fibroblast-populated collagen type I gels upon the “education” of fibroblasts with the respective control media and the CM of CRC cells for four days. Collagen gels with rhTGFβ1-activated fibroblasts showed a decreased area when compared with the control (DMEM alone), demonstrating that activation with rhTGFβ1 increased the contraction capacity of CCD-18Co fibroblasts. CM from HCT116 cells (siCTRL and siKRAS) had no effect on the collagen area when compared with the DMEM control. Contrarily, the fibroblast-populated collagen gels in CM from LS174T cells (both siCTRL and siKRAS) showed an increased area when compared with the DMEM control, indicating a decreased fibroblast contraction capacity (Figure 3A,B).

**Figure 4:**
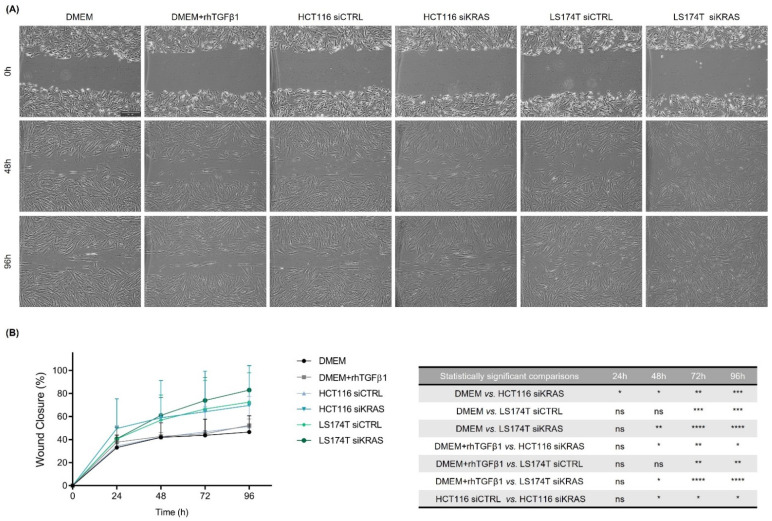
**Conditioned media from HCT116 siKRAS and LS174T siCTRL and siKRAS cells increased the migration capacity of fibroblasts.** When a confluent monolayer was formed, a wound was made and control media (DMEM and DMEM+rhTGFβ1) and CM from HCT116 and LS174T (siCTRL and siKRAS) cells were added to the respective condition. (**A**) Representative micrographs of all conditions at timepoints 0 h, 48 h and 96 h. All images were acquired using a 5 × objective. The scale bar corresponds with 500 µm. (**B**) Quantification of the percentage of wound closure in each timepoint and the respective statistically significant differences. No differences were found in the migration of fibroblasts cultured in DMEM alone or in DMEM+rhTGFβ1. CM from HCT116 siKRAS cells significantly increased the fibroblast migration capacity when compared with DMEM alone at all timepoints, and from timepoint 48 h forward when compared with DMEM+rhTGFβ1 and with the CM of HCT116 siCTRL cells. The CM of LS174T cells (siCTRL and siKRAS) increased the fibroblast migration capacity when compared with DMEM and DMEM+rhTGFβ1 (siCTRL at 72 h and 96 h; siKRAS from 48 h forward). Three independent biological replicates were performed. Values were plotted as mean ± SD of three biological replicates and statistical significance was evaluated using a two-way ANOVA considering repeated measures by both factors with Tukey’s multi-comparison test (* *p* ≤ 0.05; ** *p* ≤ 0.01; *** *p* ≤ 0.001; **** *p* ≤ 0.0001; ns: not significant).

To evaluate the impact of the CRC cell-secretome on the migration capacity, we resorted to a wound-healing assay in which we evaluated the percentage of wound closure during the course of four days. Surprisingly, the results showed no differences in the migration of the fibroblasts cultured in DMEM alone or in DMEM+rhTGFβ1. However, the CM from HCT116 siKRAS cells significantly increased the migration capacity when compared with DMEM alone at all timepoints, and from timepoint 48 h forward when compared with DMEM+rhTGFβ1. This effect was also verified when comparing with the migration rate induced by the CM from HCT116 siCTRL cells (from timepoint 48 h forward), demonstrating a KRAS dependency. On the contrary, independently of KRAS, the fibroblasts with CM of LS174T cells also migrated faster than DMEM and DMEM+rhTGFβ1 (siCTRL at 72 h and 96 h; siKRAS from 48 h forward) (Figure 4A, B).

In summary, these results showed that the CM from HCT116 cells had no impact on the contraction capacity of fibroblasts, however, the CM from KRAS silenced cells was able to increase fibroblast-migratory capacity. In turn, the CM from LS174T cells decreased fibroblast-contraction capacity while increasing their migratory ability, being both effects independent of KRAS.

## 4. Discussion

Cancer cells and fibroblasts within the TME are known to establish a crosstalk that is essential for tumor progression and malignancy. One of the routes involved in this interaction occurs via secreted factors by both cell types that are able to modulate each other properties [15,16]. The impact of cancer cell-specific alterations—namely, KRAS oncogenic activation—on the regulation of this crosstalk is poorly explored. We recently reported that approximately 2/3 of mutKRAS-associated proteome is regulated by fibroblast-secreted factors [21], impacting processes such as cell invasion [22]. Herein, we addressed how mutKRAS impacts fibroblast properties. Our data revealed that, in contrast to what has previously been described regarding the capacity of mutKRAS cancer cells to modulate the tumor immune microenvironment, the pro-tumorigenic features of fibroblasts are mainly regulated by CRC cells independently of oncogenic KRAS. Nevertheless, we acknowledge the limitation of having performed all the studies on a single normal colon fibroblast cell line. The validation of our findings on a different normal colon fibroblast cell line or in colon-derived primary fibroblasts is fundamental to fully support our observations.

In analogy to tissue myofibroblasts, the major features of the “activated” state of fibroblasts rely on the expression of activation markers, the acquisition of contraction and migration capacities and a secretory phenotype involving the production of growth factors and cytokines, ECM components and remodeling enzymes [15,16]. Among the factors that can activate fibroblasts, TGFβ is recognized as a major inducer [25,26,27]. TGFβ signaling is related to the expression of the cytoskeleton protein α-SMA, one of the most widely accepted fibroblast-activation markers as well as with ECM construction and remodeling activities [16,27,28,29]. In CRC, enhanced TGFβ signaling has been identified and associated with a stromal signature that correlates with a poor prognosis [30], and stromal TGFβ has been shown to be essential for metastasis formation [31]. Our results showed that the activation of fibroblasts with rhTGFβ1 resulted in increased α-SMA protein expression as well as the upregulation of *Fn1*, *Col1A1* and *Col1A4* and, even though not reaching a statistical significance, there was a tendency to upregulate *MMP2* and *3*. It was also capable of increasing the collagen contraction capacity, but failed to show an effect on the migration rate. In line with these results, the HCT116 cells (both siCTRL and siKRAS) were shown to produce high levels of TGFβ1; the CM from these cells were capable of increasing the protein levels of α-SMA as well as contributing to the increased levels of TGFβ1 detected upon the stimulation of fibroblasts with these CM, possibly through an autocrine positive feedback loop. The production of high levels of TGFβ1 by this cell line along with the consequent increase in fibroblast production of this growth factor and increased α-SMA expression have also been reported by other authors [29]. Moreover, despite the lower levels of TGFβ1 secreted by LS174T siCTRL cells (relatively to HCT116 cells) this CM was also able to increase the levels of α-SMA, thus suggesting that other factors substituted TGFβ1 in the activation of fibroblasts. This observation was further supported by the fact that the CM of LS174T siKRAS cells, despite having similar levels of TGFβ1, did not increase α-SMA expression.

HGF is a well-known fibroblast-secreted pro-invasive factor in various solid cancer models [23,32,33] and our previous work showed that it can drive HCT116 and LS174T cells invasion, in a mutKRAS-dependent way [22]. In addition to its pro-invasive role, stromal-derived HGF has been reported to promote the adhesion of CRC cells to endothelial cells, facilitating metastasis [34] as well as to underlie the resistance mechanisms to RAF inhibitors in a melanoma model [35] and to EGFR targeting in different cancer types [32,33]. Moreover, it has also been reported to induce the expression of fibroblast-activation markers in breast [24] and gastric cancer [36] models. Herein, the HGF quantification results showed that, despite not producing this growth factor, HCT116 and LS174T cell-secreted factors prompted the fibroblasts to increase HGF secretion, an effect that was independent of KRAS. Our data also demonstrated that HGF production was independent of TGFβ1-activation. TGFβ1-targeting therapies are being developed in an attempt to interfere with CAF activation and signaling [37]. However, according to our data, it is likely that TGFβ1 targeting will not be sufficient to abolish HGF production that, as already mentioned, is essential to drive cancer cell invasion. The identification of the cancer cell-derived factors triggering HGF production by fibroblasts deserves further attention.

ECM remodeling is a major fibroblast-instructed activity that impacts cancer cell invasion [38,39]. TGFβ-activated fibroblasts increase their production of fibronectin and collagen as well as of MMPs, thus impacting ECM deposition and degradation, respectively [27,29]. Our results showed that rhTGFβ1 was sufficient to upregulate the *Fn1*, *Col1A1* and *Col4A1* expression in CCD-18Co fibroblasts. However, the CM from CRC cells—particularly from HCT116, which presented high levels of TGFβ1—did not affect the expression of the ECM components analyzed. Of note, using in vitro and in vivo models of rectal cancer, mutKRAS was recently shown to downregulate the expression of ECM components, such as *Fn1,* in fibroblasts. However, this effect was shown to require epithelial cells-fibroblasts direct co-cultures [14], a condition that was not performed in this work. Nonetheless, our data reinforced these findings by showing that mutKRAS had no impact on the CRC cell-secreted factors that modulate the production of ECM components by fibroblasts. The same observation is likely to extend to the regulation of the expression of *MMPs*. TGFβ1 has been reported to upregulate the expression of *MMPs* in CRC tissue-isolated fibroblasts (particularly *MMP2*, *3*, *7*, *9*, *14*, *15*, *16* and *28*) [29]. In addition, the incubation of CRC tissue-isolated fibroblasts with CM from HCT116 cells was previously shown to increase the fibroblast expression of *MMP2, 3* and *14* [29]. In contrast with these observations, in our work the stimulation of CCD-18Co naïve fibroblasts with rhTGFβ1 failed to significantly upregulate the expression of the *MMPs*, although a tendency to upregulate *MMP2* and *3* was observed. Contrarily, the CM from the CRC cells (HCT116 siCTRL/siKRAS and LS174T siCTRL) led to a downregulation in the expression of *MMPs (MMP1, 2* and *3* in HCT116 and *MMP2* as well as *MMP14* in LS174T) in CCD-18Co fibroblasts. The different origins and states of activation of the fibroblasts used in our work compared with the work of Hawinkels and colleagues [29] may explain the discrepancy of the results: whereas we began with normal-like fibroblasts, which were then stimulated with CRC cells-CM, they [29] started from cancer-isolated fibroblasts. Additionally, out of all the MMPs analyzed, we could not detect *MMP9* expression in any of the conditions. According to the literature, *MMP9* expression seems to require the direct co-culture of fibroblasts with cancer cells [40] and a 3D culture system [41], in opposition to the monolayer cultures used in our study.

TGFβ1 (along with increased α-SMA expression) has also been reported to increase the fibroblast–collagen contraction capacity in different models [28,42], and an increased α-SMA expression was demonstrated to be sufficient to enhance the contractile activity of different fibroblast models [43]. Our results regarding the collagen contractility of fibroblasts stimulated with rhTGFβ1 corroborated these observations. However, a different scenario was found when the fibroblasts were treated with the CM of cancer cells. Specifically, the fibroblasts treated with the CM from LS174T cells presented slightly higher levels of TGFβ1 compared with the DMEM -treated fibroblasts, although a decrease in the fibroblast contraction capacity was observed. Also, no changes regarding collagen contractility were observed when the fibroblasts were stimulated with the CM of HCT116 cells (siCTRL and siKRAS) despite the observed increase in TGFβ1 levels and significant upregulation of α-SMA expression relatively to the DMEM-treated fibroblasts. The migratory capacity of the fibroblasts was also differentially regulated among the different conditions tested. For instance, DMEM alone, DMEM+rhTGFβ1 and the CM from the HCT116 siCTRL cells did not promote any changes to the fibroblast migration capacity. Notably, the CM from the HCT116 KRAS silenced cells caused a significant increase in the migratory capacity of the fibroblasts, demonstrating that mutKRAS in HCT116 negatively regulates fibroblast migration. This KRAS-dependent effect was cell line-specific, as the CM from LS174T siCTRL and siKRAS were equally capable of increasing fibroblast migration. Interestingly, other authors have shown that CM from KRASV12-transformed mouse colon cells increased the migration capacity of fibroblasts through the action of heparin-binding epidermal growth factor-like growth factor (HB-EGF) despite having no effect on α-SMA expression [44]. These results, therefore, raise the question of whether any of the KRAS-orchestrated effects on fibroblasts are solely cell line-dependent or if they could be mutation-specific.

**Figure 5:**
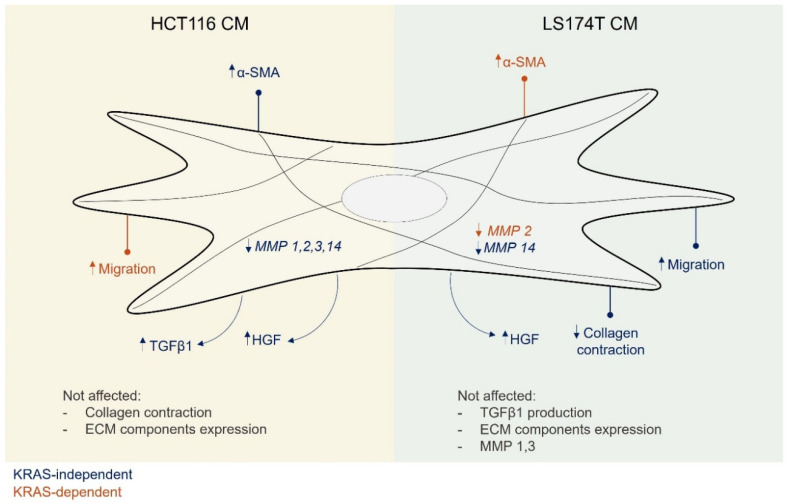
**Conditioned media from HCT116 and LS174T cells modulated different aspects of the fibroblasts phenotype, mostly independently of KRAS**. Secreted factors from HCT116 cells were able to increase fibroblast α-SMA expression and TGFβ1 secretion as well as to downregulate MMPs expression. Secreted factors from LS174T cells led to an increase in fibroblast migration and a decrease in collagen contraction capacities as well as the downregulation of *MMP14* expression. Independent of KRAS, secreted factors from both cell lines increased the levels of fibroblast-secreted HGF. A KRAS dependency was only found regarding the capacity to regulate migration, in the case of HCT116 cells, and α-SMA and *MMP2* expression in the case of LS174T cells.

Overall, the discrepancies between the TGFβ1-modulated and cancer cell CM-modulated fibroblast properties found throughout our study highlighted the potential that other factors present in the CM of the cancer cell lines could skew the fibroblast properties towards different states or subpopulations. For instance, in pancreatic cancer, tumor-secreted TGFβ and IL1 have been shown to underly the formation of two distinct fibroblast subpopulations, myofibroblastic CAFs and inflammatory CAFs, respectively [45]. Likewise, as each cancer cell line induced distinct alterations on fibroblast features (summarized in Figure 5), the study of the composition of the CM from these cell lines (growth factors, cytokines and extracellular vesicles) could help to gain further insights into the mechanisms governing fibroblast heterogeneity within and between tumors.

In summary, our study demonstrated that mutKRAS does not play a major and general regulatory role of the secreted factors that modulated the analyzed fibroblast properties. More so, each cancer cell line was able to modulate most of the fibroblast features in a cell line-dependent manner. In this scenario, mutKRAS played a secondary role by regulating discrete features in each cancer cell-educated fibroblast population.

## 5. Conclusions

The results obtained in this work raise a clinically relevant point regarding the response to the recently approved KRAS-targeted therapy and the search for combinatorial treatments to improve the response to KRAS inhibition: cells that survive KRAS inhibition are still able to promote pro-tumorigenic features in fibroblasts. The capacity revealed by KRAS-silenced CRC cells to induce fibroblast migration and activation as well as the secretion of the pro-invasive factor HGF and the immunosuppressive and epithelial-to-mesenchymal inducer TGFβ1 is noteworthy. Therefore, there is a strong possibility that KRAS-inhibited cancer cell-educated CAFs support a high degree of tolerance to KRAS inhibition observed in tumors. More so, our study opens a new window of opportunity to improve responses to KRAS inhibition by understanding whether and how the cancer cell-educated fibroblasts contribute to support tolerance and resistance to KRAS inhibition and how we can therapeutically explore this potential vulnerability.

## Data Availability

The data that support the findings of this study are available from the corresponding author upon reasonable request.

## Supplementary Materials

The following supporting information can be downloaded at: https://www.mdpi.com/article/10.3390/cells11162490/s1, Figure S1: Representative Western blots showing efficient KRAS silencing; Table S1: qRT-PCR probes used in this work.
