# Supplementary material for: Modulation of Fibroblast Phenotype by Colorectal Cancer Cell-Secreted Factors Is Mostly Independent of Oncogenic KRAS
Source: Cells. 2022 Aug 11;11(16):2490. doi: 10.3390/cells11162490 (PMC9406506; doi:10.3390/cells11162490)
Supplement: Supplementary file 1 [file cells-11-02490-s001.zip › cells-1841119-supplementary.pdf]

Supplementary Material

Supplementary Table S1. qRT-PCR probes used in this work

Gene	Reference	Manufacturer
MMP1	Hs00899658_m1	Thermo Fisher Scientific
MMP2	Hs01548727_m1	Thermo Fisher Scientific
MMP3	Hs.PT.58.14758187	Integrated DNA Technologies
MMP9	Hs00957562_m1	Thermo Fisher Scientific
MMP14	Hs00237119_m1	Thermo Fisher Scientific
FN1	Hs.PT.58.40005963	Integrated DNA Technologies
Col1A1	Hs.PT.58.15517795	Integrated DNA Technologies
Col3A1	Hs.PT.58.40254063	Integrated DNA Technologies
Col4A1	Hs.PT.58.15679435	Integrated DNA Technologies
GAPDH	Hs.PT.39a.22214836	Integrated DNA Technologies

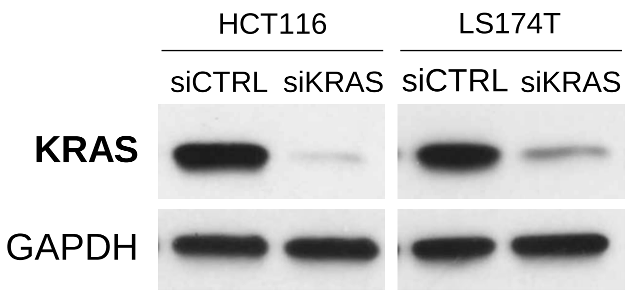

Supplementary Figure S1. Representative Western Blots showing efficient KRAS silencing.
